# Novel combination therapy for respiratory diseases of small ruminants: Field studies of over 10 years

**DOI:** 10.5455/javar.2023.j705

**Published:** 2023-09-30

**Authors:** Mohammad Hossein Gholami, Amin Derakhshanfar, Tahereh Gholami

**Affiliations:** 1 Faculty of Veterinary Medicine, Islamic Azad University of Kazerun, Kazerun, Iran; 2 Department of Comparative Biomedical Sciences, School of Advanced Medical Sciences and Technologies, Shiraz University of Medical Sciences, Shiraz, Iran; 3 Department of Foreign Language, Faculty of Humanities, Islamic Azad University of Kazerun, Kazerun, Iran

**Keywords:** Small ruminant, respiratory disease, combination-therapy

## Abstract

**Objective:**

This study aimed to evaluate a new drug combination for small ruminant respiratory diseases to find a better treatment protocol for the potential replacement of older methods.

**Materials and Methods:**

A total of 6,886 animals received common respiratory disease therapies out of 15,845 animals that had respiratory disorders. The new combination therapy technique treated the remaining animals (8,968). The animals were given an oral suspension of triclabendazole or levamisole at an initial dosage of 0.2 ml/kg body weight (BW). The following day, 0.2 mg/kg of 1% ivermectin was subcutaneously administered. Then, on the third and fifth days of treatment, a subcutaneous injection of 30 mg/kg BW of florfenicol (30%) was administered. The survival and recovery rates for both groups were tracked throughout a 6-month period of observation. Postmortem and histopathological signs were also assessed.

**Results:**

In the group of the novel combination therapy, group A, clinical, postmortem, and histopathological signs were significantly reduced compared to group B. Clinical signs and mortality in group A were 90% and 93% lower than in group B, respectively. Animals that received the new combination therapy were healed of their disease and stayed immune for 6 months.

**Conclusion:**

This novel therapy demonstrated significant efficacy against respiratory diseases in a 10-year field study. The paper proved that the protocol introduced could be a new therapeutic approach.

## Introduction

Small ruminant flocks worldwide commonly face respiratory diseases, making them one of the most prevalent issues in their farming, which causes reduced productivity and death, resulting in financial loss [1–3]. The problem is also a welfare concern for animals, but the causes seem diverse. It mostly results from damaging weather conditions, stress, and bacterial and viral infections [3–5]. The most common bacterial agents that can cause respiratory disorders include *Mannheimia haemolytica*, *Mycoplasma ovipneumoniae*, and *Mycoplasma argini*. Among viral agents, Parainfluenza-3 virus, reovirus, respiratory syncytial virus, and ovine adenovirus type 6 are frequently reported [3,6]. *Mycoplasma* and viral agents cause mild respiratory disorders; however, bacterial involvement, e.g., *M. haemolytica*, results in high mortalities and prominent clinical signs [6,7]. The lifestyle of nomadic pastoralists is similar to that of humans. Also, the lung structure of sheep is similar to the lung structure of humans [8], so it is beneficial to use sheep respiratory disease algorithms to model human respiratory disease.

Levamisole is a well-established antiparasitic medication that has been used for a considerable period of time to treat and prevent various parasitic infections [9]. It was discovered as an antiparasitic medication in 1966 and was first employed against *Trichuris trichiura*, *Ascaris lumbricoides*, and hookworms in humans and animals [10]. After a decade of continuous use, it is one of the well-known imidazothiazole families with little drug resistance [11]. Many studies have examined levamisole as an immunostimulant against viruses [12,13]. Scientists used levamisole in adjuvant and supplementary therapy [13]. Interestingly, many studies have indicated the protective effect of levamisole on foot and mouth disease (FMD) in animals. The immunostimulatory potential of levamisole was shown in buffaloes vaccinated with FMD serotypes O, A, and SAT2 [14]. Levamisole administration with the FMD vaccine promoted humoral and cell-mediated immunity in vaccinated sheep 8 and 14 weeks after levamisole administration [15].

Florfenicol is a thiamphenicol analog with a fluorine atom replacing the hydroxyl group in the molecule. Florfenicol is thus effective against bacteria that produce acetyltransferase and bacteria resistant to chloramphenicol. Because florfenicol lacks a nitro group, it is not linked to aplastic anemia like chloramphenicol [16]. It is licensed to treat *M. hemolytica* in bovine and sheep respiratory diseases [17–19]. Florfenicol acts as a bacteriostatic agent, inhibiting bacterial protein synthesis by binding to the 50S subunit of the bacterial ribosome [20].

Since it was first discovered in 1967, ivermectin has been a wonder drug for treating various diseases, including bacterial and viral ones. Ivermectin has various mechanisms, one of which is immunomodulation. It activates neutrophils, increasing C-reactive protein and interleukin-6 levels [21]. Ivermectin is believed to function by blocking the import of viruses and host proteins into the nucleus. It exerts its antiviral effect by inhibiting the import of viral interface inner membrane peptidase subunit 1, which is essential for most RNA viruses during infection, thereby enhancing the antiviral response [22].

In the early 1980s, triclabendazole was initially employed to treat *Fasciola hepatica* infections in animals. Over time, it has become the leading antifluke medication available, largely owing to its exceptional effectiveness against immature flukes [23]. In recent times, triclabendazole has been employed for the treatment of human cases of fascioliasis, and it has emerged as the preferred choice of treatment for this infection in humans as well [23].

This study was based on a large study size and aimed to evaluate the effect of a specific combination therapy on the treatment of respiratory diseases in goats and sheep.

## Materials and Methods

This study was approved by the Ethics Committee of the Islamic Azad University (Code: IR.IAU.VETMED.REC.1399.301).

**Figure 1.**
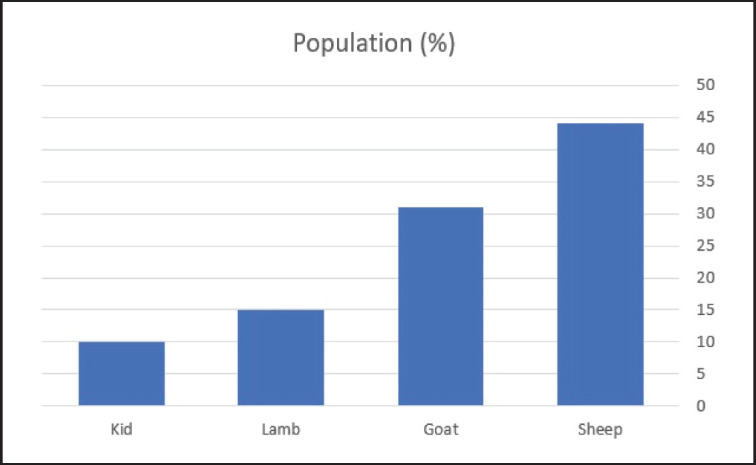
Population of cases.

This study was mainly based on the flocks referred to our veterinary clinic in Baladeh city, Fars province, Iran, over the course of 10 years (Figure 1). First, the clinical signs were examined, including cough, abnormal sound of the lung, and nasal discharge and records were made in their documents. After the identification of the ill animals, two routes for treatment were decided: one with the studied combination therapy and the other with different treatments. The new method of combination therapy included the prescription of Triclabendazole/Levamisole 8/75% (0.2 ml/kg BW) suspension (Royan Daru^®^, Iran) via oral route on the first day. On the second day, Ivermectin 1% (0.2 mg/kg) (Royan Daru^®^, Iran) was injected subcutaneously, followed by Florfenicol 30% (30 mg/kg BW) (Royan Daru^®^, Iran) on the third and fifth days of treatment. After 2 weeks, changes in the respiratory signs were checked in both groups, and the number of animals that had not recovered was recorded. The flocks were regularly observed over the course of 6 months. At the 6-month point, the number of mortalities in both groups was recorded. Each group consisted of 30 different flocks with different sheep and goat ratios, as well as different ages and sexes. group A, i.e., the group treated with this specific method, and group B had 8,967 and 6,886 members, respectively, for a total of 15,853 animals.

Postmortem and histopathological examinations were performed on slaughtered animals. Fifteen samples from each group were examined postmortem and submitted to the laboratory. The lung tissues were embedded in paraffin after being preserved in 10% buffered formalin (Merck, Germany). Sections of prepared blocks were trimmed into 5-mm-thick sections, deparaffinized with xylene, and rehydrated. Standard hematoxylin/eosin and Masson trichrome staining methods were followed [24].

For bacterial detection, 30 broncho-alveolar fluid samples were collected in a sterile container and submitted to the laboratory. The samples were inoculated onto prepoured sheep blood agar plates (Zistroyesh, Iran) and incubated for 24 h at 37°C. Single colonies were smeared on clean, glassy slides after incubation, and bacteria strains were identified using the Gram-staining technique.

Data obtained from two groups were analyzed by Statistical Package for Social Sciences software version 21 (IBM Corporation, USA). Differences were assessed using the independent-*t* test. A *p-*value of less than 0.5 was considered significant.

## Results

In group A, clinical signs vanished in 28 flocks after 2 weeks of treatment. In the 6-month period, only 24 mortalities happened across 8,967 animals. Also, no respiratory signs were observed during this period. In laboratory results, out of 15 samples, 8 *M. haemolytica* and 3 *P. multocida* isolates were detected. In comparison, in 4 samples, no bacteria were detected, which may represent the involvement of other types of pathogens such as viruses.

**Figure 2.**
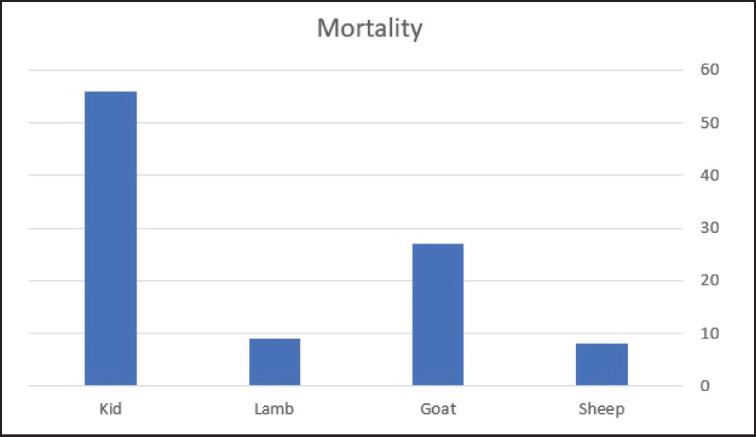
Number of mortalities per population.

**Figure 3.**
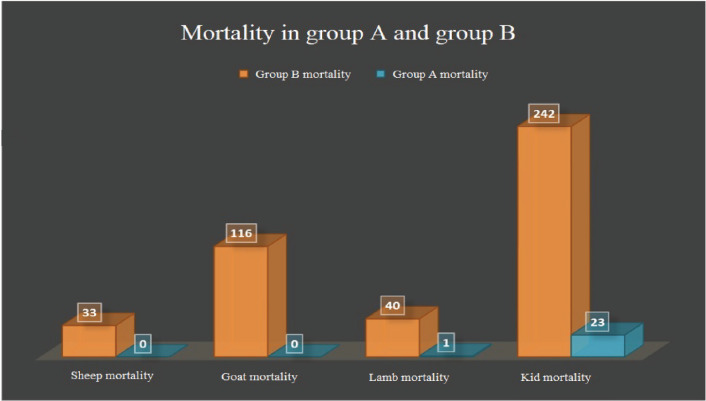
Comparison of mortality between two groups after 6 months.

In group B, 25 flocks still had clinical signs after 2 weeks of treatment, and 5 mortalities were observed in flocks with no apparent signs over the course of 6 months. Also, they showed respiratory signs during this period. The overall mortality of all 30 flocks (across 6,886 animals) was 431 cases in the mentioned period. Seven isolates of *M. hemolytica, *two isolates of *P. multocida*, and one isolate of *P. anatipestifer* were detected, while no bacteria were found in the other five samples (Figure 2 and 3).

**Figure 4.**
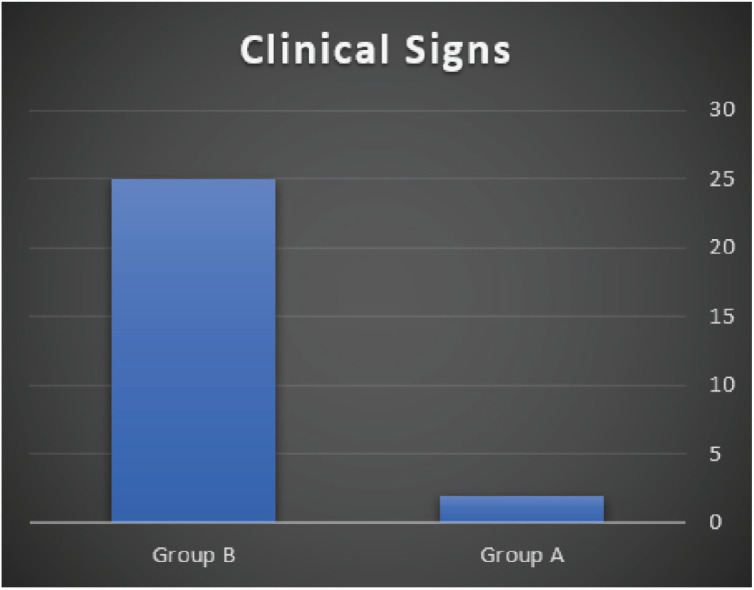
Comparison of clinical signs after 2 weeks.

In group A, the clinical signs and mortalities were lower than in group B by 90% and 93%, respectively (Figure 4).

**Figure 5.**
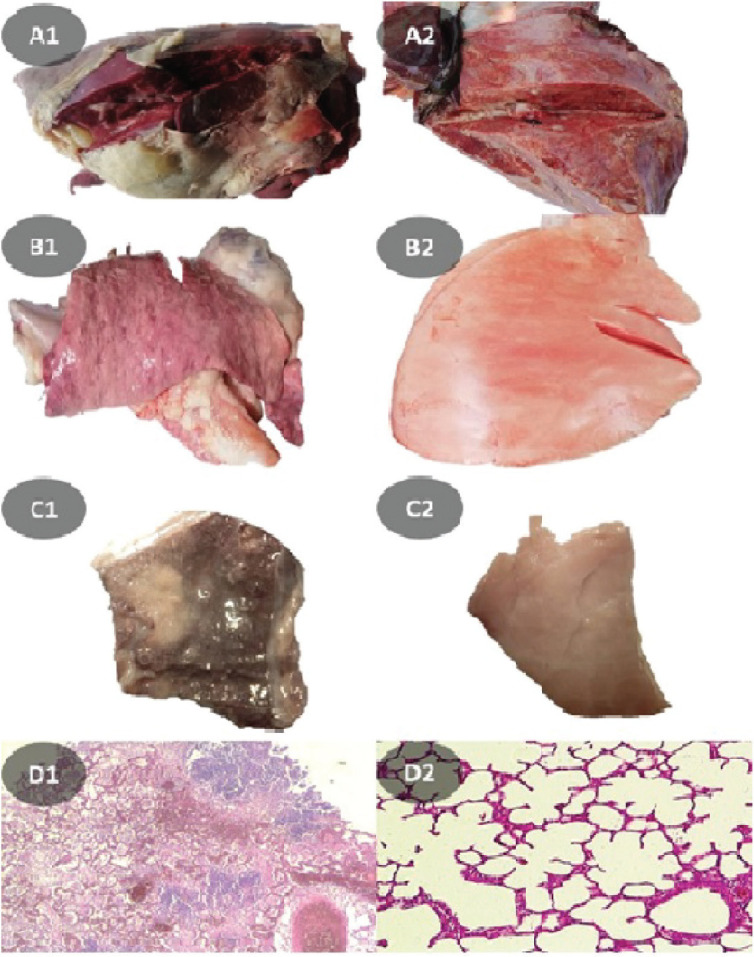
Gross aspect of untreated group lung tissue with fibrino-pleuritis (A1) and edema, bleeding, and exudate discharges (A2). B. Apparently, nearly normal lung tissue of group A. C. Sub-gross aspect of lung tissue samples from group B (C1) and group A (C2) groups. D. H&E staining of lung sections from group B (D1) and group A (D2) at magnification of 100×.

Although in histopathological evaluations of group B lung tissues, pulmonary edema, bleeding, purulent exudate, and fibrino-pleuritis were observed, group A showed normal structure with no apparent lesions. In postmortem findings, mild inflammation to severe necrosis was evident (Figure 5).

## Discussion

In this study, 70% of the samples (21/30) were infected with agents from the Pasteurellaceae family, with 15, 5, and 1 from *haemolytica*, multocida, and anatipestifer strains, respectively. In nine other cases, no bacterial agent was found, suggesting their infection could be due to viral involvement. The new combination therapy has been a very effective treatment for sheep and goat respiratory disease over the years of its application. Also, small ruminants that had received this treatment would be immune to any respiratory disease for 6 months.

Ivermectin is a rare drug that can affect many pathogenic agents, including viruses, bacteria, and parasites. Ivermectin had an inhibitory effect against *S. aureus* isolates in the study of Ashraf et al. [25]. Bentounsi et al. [26] showed that ivermectin can treat respiratory disease in Algerian sheep from nematode infestation. Both of these studies are in line with the results of this study; however, due to the discovery of the antibacterial effect of ivermectin, more studies are needed to further approve the use of ivermectin to treat bacterial agents of respiratory disease. The study of Taylor et al. [27] evaluated the potential antimicrobial activity of ivermectin and two other agents against scabies. Their results showed that ivermectin can eliminate bacteria and prevent secondary infections common in scabies [27]. However, it cannot prove antibacterial activity in respiratory diseases. In that regard, methicillin-resistant *S. aureus* (MRSA) is a common bacterium causing respiratory disease, and in the study of Tan et al. [28] an ivermectin-derived compound, D4, was proven to eliminate MRSA. Ivermectin can influence the host’s immune response and its antiparasitic actions. It also has antiviral properties [29]. Also, human ivermectin usage is vastly studied, especially with the recent COVID-19 outbreak. Ivermectin was used to treat COVID-19 virus-infected Vero/hSLAM cells in an *in vitro* investigation by Caly et al. [30] after 48 h, a 5,000-fold reduction—eliminating nearly all virus particles—was recorded [30].

The immunopotentiation effect of levamisole on various animals is well documented. In the study of Darwish and Eldakroury [31], when levamisole was injected subcutaneously into male Barki lambs, its immunostimulatory potential was evident. In addition, treatment groups appear to have gained more weight. Examining the key characteristics of blood samples after co-administration of levamisole with various sheep vaccines revealed both long- and short-term immune-protective stimulation of levamisole [32,33]. Levamisole is thought to have a favorable effect on both large and small ruminant animals that affects several aspects of the pre and postparturition period and neonatal immunology [34]. After administering oral levamisole treatment (2 mg/kg) for 28 days in 30 herds of 2-day-old Holstein calves, no significant differences were observed in the key indicators of packed cell volume, white blood cell count, differential leukocyte count, total serum protein, and illness occurrence between the treatment groups and the control group when compared to the effects of injection. However, there were noticeable differences in the levels of gamma globulin, monocytes, and neutrophils compared to the control group [35]. Aside from animal studies, levamisole has been studied in human research. In a recent study, levamisole had few side effects and helped lower the risk of relapse in steroid-sensitive types of nephrotic syndrome [36].

The potency of florfenicol in treating respiratory diseases is also well-studied. In one study on Sakiz sheep, various antibiotics were used to treat *M. hemolytica*. Among all therapeutic agents, florfenicol was the only antibiotic effective against all isolates [37]. Also, in another study, common respiratory pathogens in sheep and goats were tested against various common antibiotics (florfenicol, co-amoxiclav, ceftiofur, tetracycline, and ciprofloxacin). Among ovine and caprine isolates, florfenicol was the most potent antibiotic against *M. hemolytica* and *P. multocida* [38].

## Conclusion

The presented new protocol is very effective against respiratory pathogens. Also, this new protocol guarantees 6-month respiratory disease immunity; however, further studies are needed to explain the precise mechanism.

## List of Abbreviations

BW, Body weight; COVID-19, Coronavirus disease of 2019; FMD, Foot and mouth disease; H&E, Hematoxylin and eosin; MRSA, Methicillin-resistant *S. aureus*; WBS, Whole blood serum.
